# Low vitamin D levels are associated with high viral loads in patients with chronic hepatitis B: a systematic review and meta-analysis

**DOI:** 10.1186/s12876-019-1004-2

**Published:** 2019-06-11

**Authors:** Ye-Chao Hu, Wei-Wei Wang, Wei-Yun Jiang, Chun-Qing Li, Jian-Chun Guo, Yun-Hao Xun

**Affiliations:** 1 Department of Liver Diseases, Hangzhou Sixth People’s Hospital/Xixi Hospital of Hangzhou, Zhejiang University of Traditional Chinese Medicine, 2 Hengbu Road, Hangzhou, 310023 China; 2 The First People’s Hospital of Xiaoshan District, 199 Shixin South Road, Hangzhou, 311200 China

**Keywords:** Hepatitis B virus, Hepatitis B, chronic, Vitamin D deficiency, 25-hydroxyvitamin D, Meta-analysis

## Abstract

**Background:**

Previous studies have investigated the vitamin D status in patients with chronic hepatitis B virus (HBV) infection and its relationship with HBV replication, the results however were inconsistent. The present meta-analysis was carried out to compare the vitamin D levels between patients with chronic hepatitis B (CHB) and healthy controls, and to determine whether vitamin D levels were correlated with HBV viral loads significantly.

**Methods:**

A systematic search was conducted via PubMed, Web of Science, EMBASE and the Cochrane Library to identify eligible studies until September 28, 2017. We calculated pooled mean difference (MD) and 95% confidence intervals (CI) to quantitatively estimate the difference of vitamin D levels between CHB patients and controls. In addition, correlation between serum vitamin D levels and HBV viral loads was defined by summary correlation coefficient (r value) and the corresponding 95% CI.

**Results:**

A total of 7 studies involving 814 CHB patients and 696 healthy controls were included. A significantly decreased vitamin D levels was found in CHB patients compared with healthy controls: pooled MD (95% CI) was − 2.03 ng/mL (− 2.60, − 1.46). Latitude-stratified subgroup analysis indicated this difference was more obvious in low latitude areas, with a bigger pooled MD (95% CI) of − 2.72 ng/mL (− 4.57, − 0.87). In addition, we observed an inverse correlation between serum vitamin D levels and HBV viral loads: pooled r (95% CI) was − 0.41(− 0.54, − 0.27).

**Conclusions:**

Our results showed that vitamin D levels were lower in CHB patients than that of healthy controls and inversely correlated with HBV viral loads, although future comprehensive studies are needed to clarify the underlying mechanisms.

**Electronic supplementary material:**

The online version of this article (10.1186/s12876-019-1004-2) contains supplementary material, which is available to authorized users.

## Background

Hepatitis B virus (HBV) infection remains an important public health problem worldwide [1, 2]. Approximately one third of world’s population has been infected, and over 350 million patients are suffering from chronic HBV infection [1–3]. The clinical manifestations of chronic HBV infection include an inactive carrier state, chronic hepatitis, cirrhosis and even hepatocellular carcinoma (HCC) [4, 5]. The outcome of infection depends on the interactions between host and viral factors, including gender, age, immune response, HBV genotype, HBV DNA levels, etc. [1, 2, 6].

Vitamin D is a fat-soluble vitamin including two forms: vitamin D_3_ or cholecalciferol and vitamin D_2_ or ergocalciferol [7]. Natural sources of vitamin D in humans are mostly cholecalciferol (also called vitamin D_3_) which is synthesized in the skin from 7-dehydrocholesterol by sunlight exposure. Cholecalciferol is firstly converted by the 25-hydroxylase to 25-hydroxyvitamin D (25OHD) in the liver and further by 1α-hydroxylase in the kidney to produce bioactive vitamin D metabolite, 1,25-dihydroxyvitamin D [1,25(OH)_2_D] [7, 8]. The 1,25(OH)_2_D acts through vitamin D receptor (VDR) in a large number of tissues to carry out its classical functions, including the regulation of calcium, phosphorus, and bone mineral homeostasis. It also has several noncalcemic functions, such as immunomodulatory, anti-inflammatory and anti-fibrotic properties, in the body [7–9]. Serum vitamin D level is affected by gender, season, latitude and genetic variations related to vitamin D metabolism [10]. Although there is no consensus on optimal levels of vitamin D in serum, a 25OHD level < 20 ng/mL (< 50 nmol/L) is considered to indicate vitamin D deficiency by most experts [7, 10]. Vitamin D deficiency results in abnormalities in calcium, phosphorus, and bone metabolism. Also, it has been suggested to be involved in the pathogenesis of autoimmune disorders, type 2 diabetes, cancers and the course of several infectious diseases recently [8, 9]. With regard to liver diseases, clinical evidence has shown that patients with chronic liver diseases such as chronic hepatitis C (CHC) and non-alcoholic fatty liver disease (NAFLD) are at higher risk of vitamin D deficiency [11]. Furthermore, low serum levels of 25OHD was reported to be an independent factor of adverse outcomes in alcoholic liver diseases [12] and impaired virological response to interferon-based therapy in CHC patients [13, 14]. A meta-analysis also demonstrated high prevalence of vitamin D deficiency in CHC patients, and a better virological response in individuals with higher serum vitamin D levels or receiving vitamin D supplementation [15].

Previous study demonstrated that vitamin D deficiency is prevalent in patients with chronic HBV infection and the serum 25OHD levels are inversely correlated with HBV viral loads [16]. Since then, several additional studies have assessed the vitamin D status in HBV patients and its correlation with HBV replication. However, the results of these studies were inconclusive. The aims of this meta-analysis are to evaluate the vitamin D status in CHB patients compared with healthy population, as well as the possible association between 25OHD and HBV DNA levels.

## Methods

This meta-analysis was conducted in accordance with Preferred Reporting Items for Systematic Reviews and Meta-Analyses (PRISMA) guidelines (Additional file 1: Data S1) [17].

### Data sources and search strategy

Two authors (Y.C Hu, W.W Wang) independently searched articles on databases including PubMed, Web of Science, EMBASE and the Cochrane Library. The keywords used in our searches are as follows: “chronic hepatitis B”, “hepatitis B”, “vitamin D”, “25OHD”, and “1,25(OH)_2_D”. The last search was performed on September 28, 2017.

### Study selection

After removing duplicate articles, two authors (Y.C Hu, W.Y Jiang) independently screened the titles and abstracts for potentially relevant studies, and reviewed the full texts when necessary. Included studies had to meet the following criteria: (1) Observational studies of all designs; (2) Data on serum vitamin D values in treatment naïve CHB patients (because both IFN-α and some nucleos(t)ide analogues may have an effect on vitamin D metabolism) [16] and healthy controls; (3) The outcome was presented as mean vitamin D values and their standard deviations in patients and controls, or correlation coefficients between serum vitamin D levels and HBV DNA levels; (4) Because HBV DNA levels in the original studies were log-transformed before analysis, Spearman’s correlation coefficient, instead of Pearson’s correlation coefficient, was applied in this study. As for exclusion criteria, reviews, case reports, conference paper, editorials and irrelevant articles were excluded. Any disagreement was resolved by discussion, together with clinical experts consultation.

### Data extraction and quality assessment

The following information was extracted from eligible articles: name of the first author, year of publication, country, study design, method used for the vitamin D assay, season of sampling, latitude, sample size, participant characteristics (gender, age), virological parameters (HBV genotype, HBV DNA levels, HBeAg status), serum vitamin D levels, prevalence of vitamin D deficiency in CHB groups, and correlation coefficients between serum vitamin D and HBV viral loads.

The methodological quality of studies was assessed by two authors (Y.C Hu, W.Y Jiang) independently using the Newcastle–Ottawa Scale (NOS) [18]. In general, > 7 scores are regarded as a high quality while 5–7 scores are regarded as a moderate quality [19]. Any disagreement between authors was resolved as described above.

### Data synthesis and statistical analysis

Mean difference (MD) with 95% confidence interval (CI) was used to determine the difference of serum vitamin D levels between patients and control groups. When referring to the association between vitamin D and HBV viral loads, all the correlation coefficients (r values) were firstly transformed to Fisher’s Z values using Fisher’s r to Z transformation (Formula 1). Standard errors of correlation coefficients were calculated with Formula 2 and Formula 3. Then summary Fisher’s Z value was calculated with the generic inverse variance method. The result was finally transformed back to the original correlation coefficient metric (Formula 4) [20, 21]. Formulas 1–4 are shown in (Additional file 2: Figure S1).

Heterogeneity between studies was estimated using Cochran *Q* test and *I*^2^ statistics. *P* < 0.05 or *I*^2^ > 50% indicated significant heterogeneity, and these data were analyzed with random-effects model to accommodate diversity. Otherwise, the fixed-effects model was applied [22, 23].

For any heterogeneity, sensitivity analyses were performed to indentify individual study’s effect on pooled results and test the reliability of results. Funnel plots and Egger’s test were used to assess the potential presence of publication bias. All statistical analyses were conducted using Stata version 12.0 software (Stata Corp LP, College Station, Texas) and R, version 3.3.1 for Windows (http://www.r-project.Org/).

## Results

### Search results and study selection

**Fig. 1:**
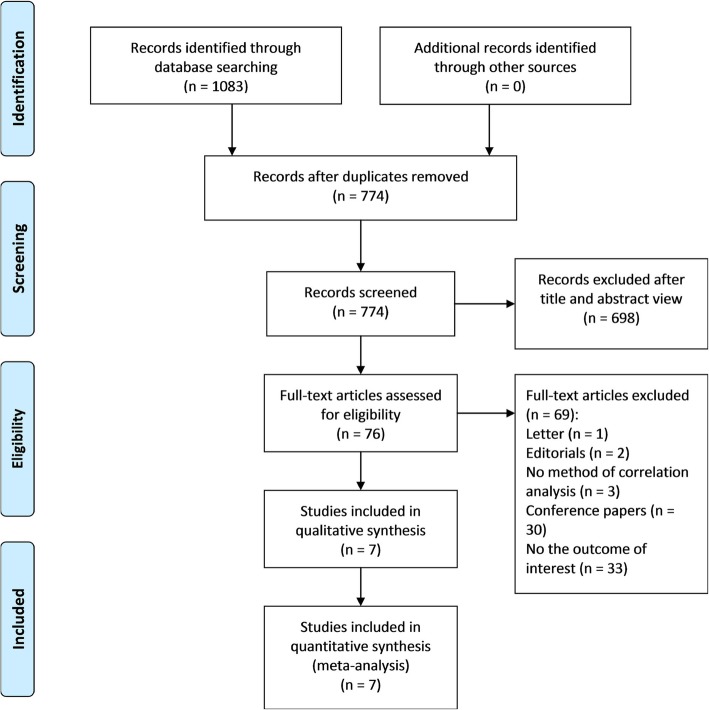
PRISMA flow diagram of the study selection process

Our search identified 1083 articles, of which 309 were removed as duplicate publications and 698 were excluded after screening titles and abstracts. Therefore, a total of 76 articles were retained for full-text review, and 69 articles were excluded for other reasons (1 letter, 2 editorials, 3 did not report the method of correlation analysis, 30 conference papers and 33 did not report the outcome of interest). Finally, 7 eligible articles involving 814 patients with HBV infection cases and 696 controls were included in this meta-analysis [24–30]. The screening process was detailed in Fig. 1.

### Study characteristics and quality assessment

The included studies were published between 2012 and 2017. All the studies were reported as full-text articles in English. 5 studies were conducted in Asia, 1 in Middle East and 1 in Europe.

All the studies were observational studies. One study [28] only involved pregnant women and one only involved men [30]. Two studies [27, 29] involved HBV patients with different clinical stages, including CHB, cirrhosis and HCC. And one study [24] involved patients with viral hepatitis B or C. Only the appropriate data of CHB patients in each study was singled out for this meta-analysis.

All but one [24] of the studies reported the proportion of gender among CHB patients, yet no gender specific level of vitamin D was available. Four studies [24, 26, 28, 29] mentioned the seasons of blood sampling but the seasons were different from each other, and no seasonally fluctuation was showed in Chen’s study which extensively addressed the seasonality of serum vitamin D [26]. Only one study [26] reported HBV genotype. Two studies [26, 29] explored the differences of vitamin D level per HBeAg status and showed paradoxical results, with another study [28] indicating a comparable frequency of vitamin D deficiency. Thus no specific subgroup analyses about aforementioned items have been done.

**Fig. 2:**
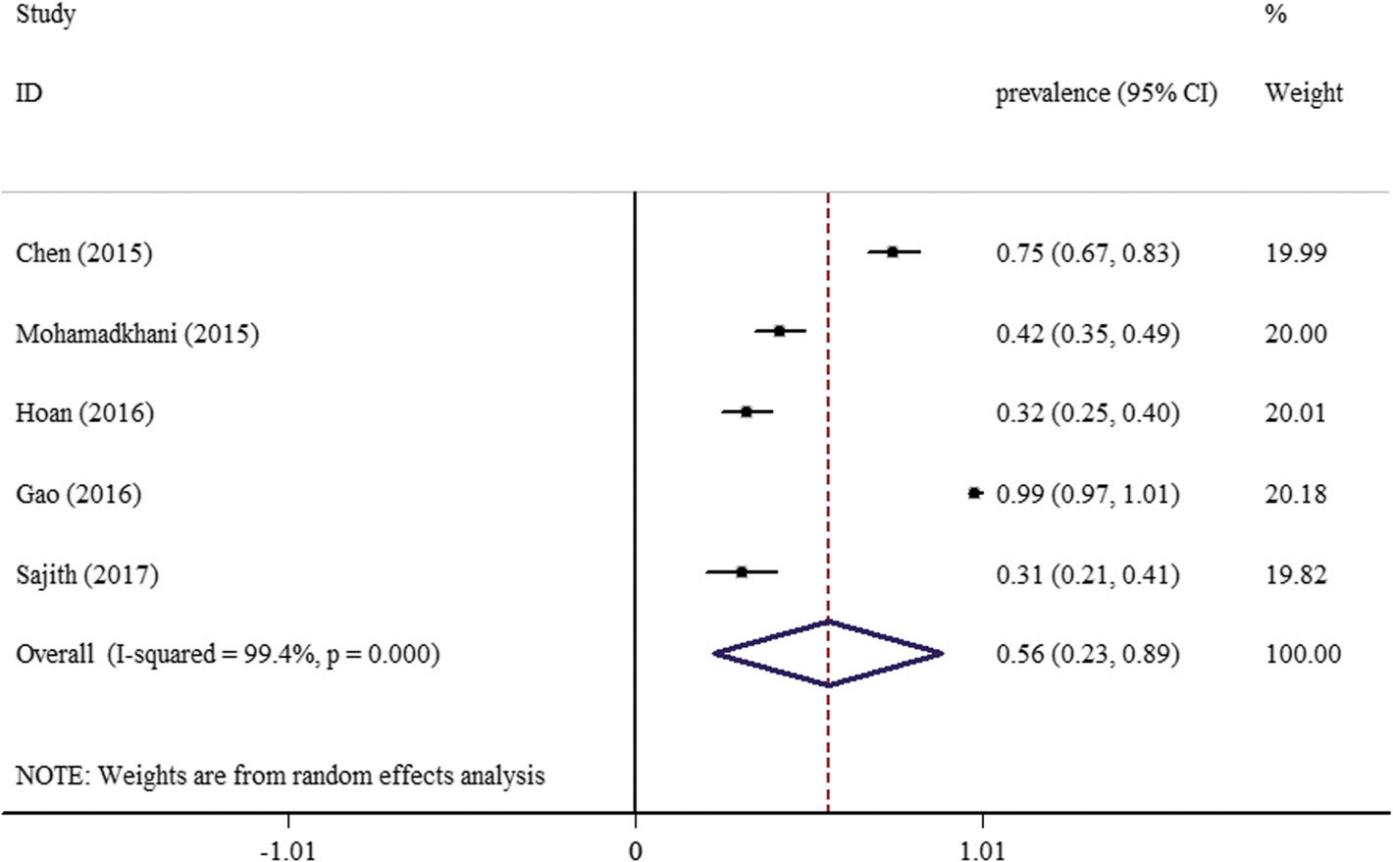
Forest plot of overall prevalence of vitamin D deficiency in patients with chronic hepatitis B

Five studies [25–28, 30] reported the proportion of patients with vitamin D deficiency (< 20 ng/mL) in CHB groups. And the pooled prevalence of vitamin D deficiency was 56% (95% CI: 23–89) (Fig. 2).

In addition, serum 25OHD was measured by different methods, including Enzyme-Linked Immunosorbent Assay, electrochemiluminescence, High Performance Liquid Chromatography Tandem Mass Spectrometry, and chemiluminescence. Even though, the interferences in this aspect are considered to be negligible, as reported by Zeng et al. [31].

Using the NOS assessment, 3 observational studies [26, 29, 30] were found to be of high-quality and 4 observational studies [24, 25, 27, 28] were labeled as moderate-quality studies. The characteristics and quality assessment of included studies are detailed in Table 1.

**Table 1:** Main characteristics of included studies

First author	Year	Country	Study design	Method	Season of sampling	Latitude	Number of CHB patients (male)	Age of CHB patients (years)^a^	Number of healthy controls (male)	Age of healthy controls (years)^a^	HBV genotype: B/C (n, %)	HBV viral loads^a^	HBeAg status: P/N (*n*, %)	Vitamin D in patients (mean ± SD)(ng/mL)	Prevalence of vitamin D deficiency in CHB patients (%)	VitaminD in controls (mean ± SD)(ng/mL)	Correlation coefficient(r)	NOS score
Iacob [24]	2012	Romania	Observational	ELISA	Autumn to spring	NA	12(NA)	NA	22 (7)	36.22 ± 13.86	NA	NA	NA	11.5 ± 6.36	NA	12.04 ± 3.99	NA	7
Mohamadkhani [25]	2015	Iran	Observational	ELISA	NA	NA	173 (118)	37 ± 9.7	NA	NA	NA	3.94 ± 1.08^c^	NA	22.19 ± 8.28	42	NA	−0.32	6
Chen [26]	2015	China	Observational	ECL	All year round	NA	128 (92)	33.57 ± 8.47	128 (95)	35.17 ± 8.02	83 (64.8)/45 (35.2)	6.38 ± 2.32^d^	91 (71.1)/37 (28.9)	16.88 ± 6.4	75	20.16 ± 5.5	−0.392	8
Hoan [27]	2016	Vietnam	Observational	ELISA	NA	NA	165 (135)	39 (18–79)^b^	122 (82)	40 (19–58)^b^	NA	5.6 (2.4–10.1)^b,c^	NA	21.2 ± 8.9	32.5	23.6 ± 9.5	−0.57^f^	7
Gao [28]	2016	China	Observational	ELISA	Winter and summer	NA	142 (0)	25.8 ± 3.5	251 (0)	26.5 ± 3.8	NA	NA	110 (77.5)/32 (22.5)	12.05 ± 3.3	98.6	13.63 ± 5.5	NG	7
Zhao [29]	2016	China	Observational	HPLC-TMS	Winter	38°3′N-39°54′N	115 (89)	51.29 ± 9.26	115 (89)	51.41 ± 9.17	NA	4.10 ± 1.80^e^	38 (33.0)/77 (67.0)	7.83 ± 3.47	NA	9.76 ± 4.36	NG	8
Sajith [30]	2017	India	Observational	CL	NA	NA	79 (79)	40.5 ± 10	58 (58)	38 ± 6.2	NA	NA	NA	23.2 ± 12.6	31	26.8 ± 8.7	NG	9

*Abbreviations*: *CL* chemiluminescence, *ECL* electrochemiluminescence, *ELISA* Enzyme-Linked Immunosorbent Assay, *HPLC-TMS* High Performance Liquid Chromatography Tandem Mass Spectrometry method, *NA* not applicable, *P/N* positive/negative, *SD* standard deviation

^a^: mean ± SD, unless noted otherwise

^b^: median (range)

Units of HBV viral loads: ^c^, log copies/mL; ^d^: log IU/mL; ^e^: log IU/L

^f^: only 63 patients were selected for analysis of the correlation between vitamin D levels and HBV viral loads

### Meta-analysis

#### Lower vitamin D levels in CHB patients

**Fig. 3:**
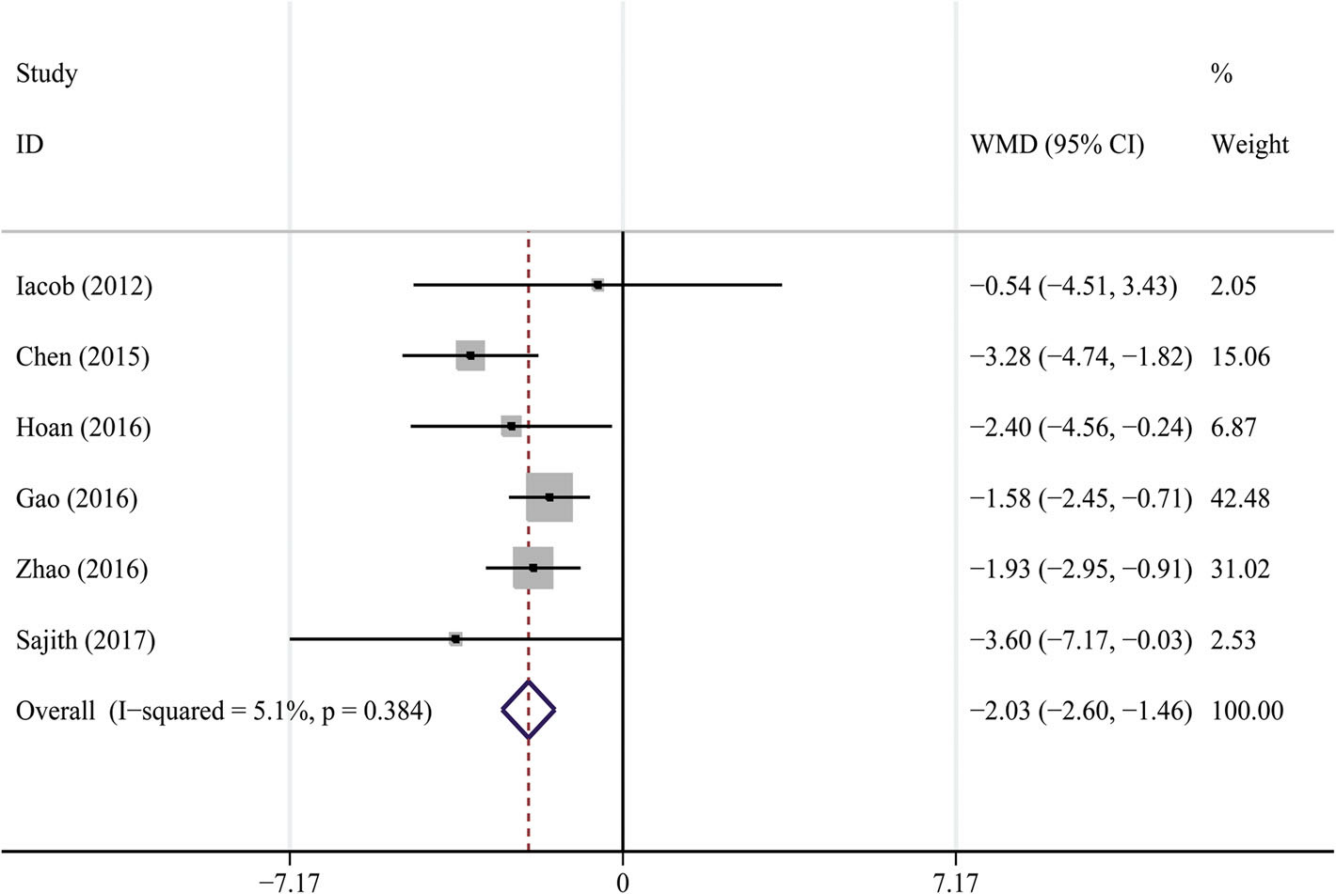
Forest plot of comparisons of vitamin D levels in CHB patients versus healthy controls

Six studies [24, 26–30] involving 641 CHB patients and 696 healthy controls were selected for this quantitative analysis. The units of 25OHD values were transformed from “nmol/L” to “ng/mL” (2.5 nmol/L = 1 ng/mL) [9] to maintain consistency. The average serum 25OHD levels were significantly lower in CHB patients than that in controls, and pooled MD (95% CI) was − 2.03 ng/mL (− 2.60, − 1.46) (Cochran *Q* test *P* = 0.384, *I*^2^ = 5.1%) (Fig. 3).

**Fig. 4:**
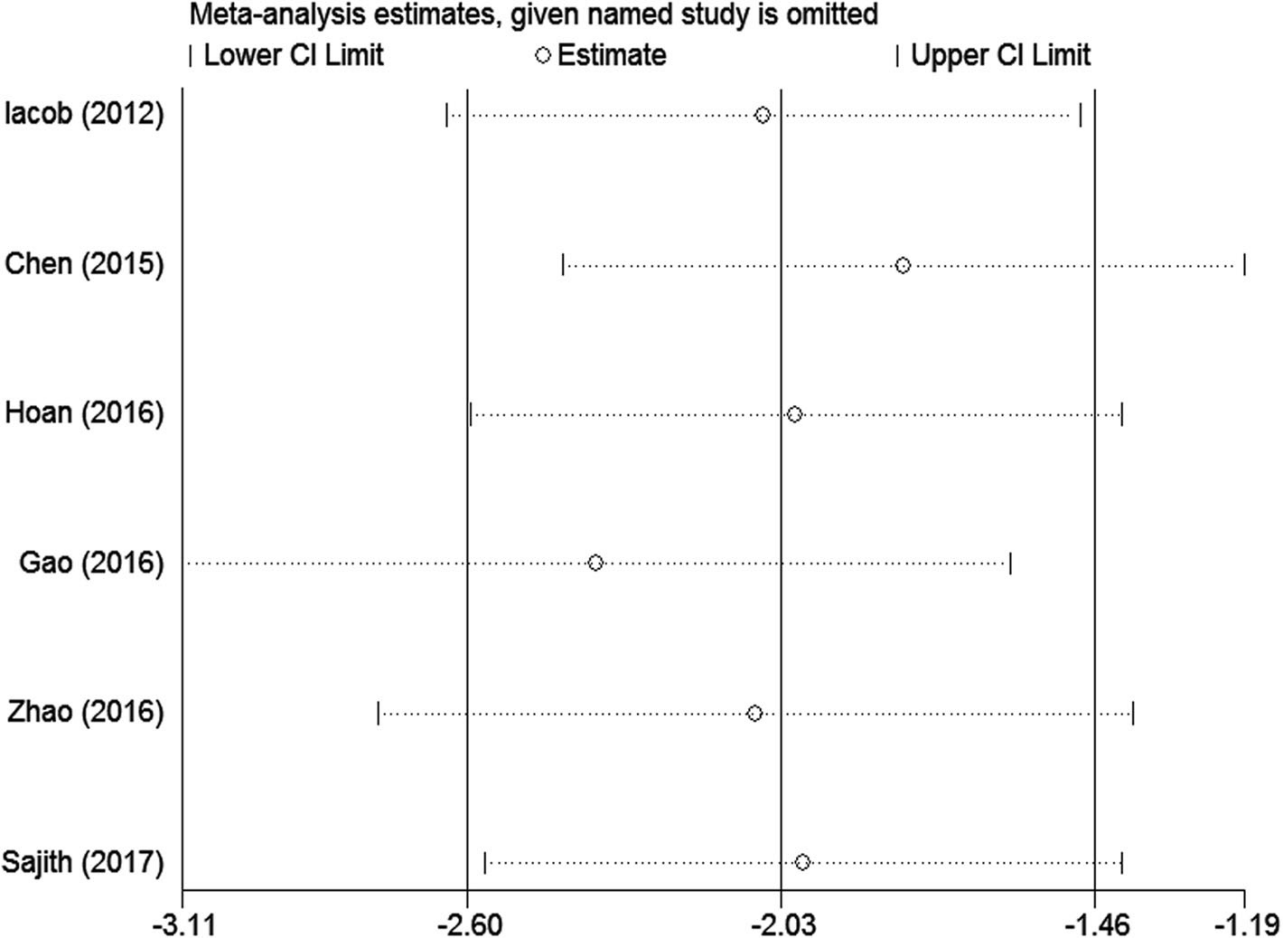
Sensitivity analysis for comparisons of vitamin D levels in CHB patients versus healthy controls

Sensitivity analysis, performed by deleting one single study each time from analysis, showed no individual study affected the result significantly, indicating that our result was robust (Fig. 4).

**Fig. 5:**
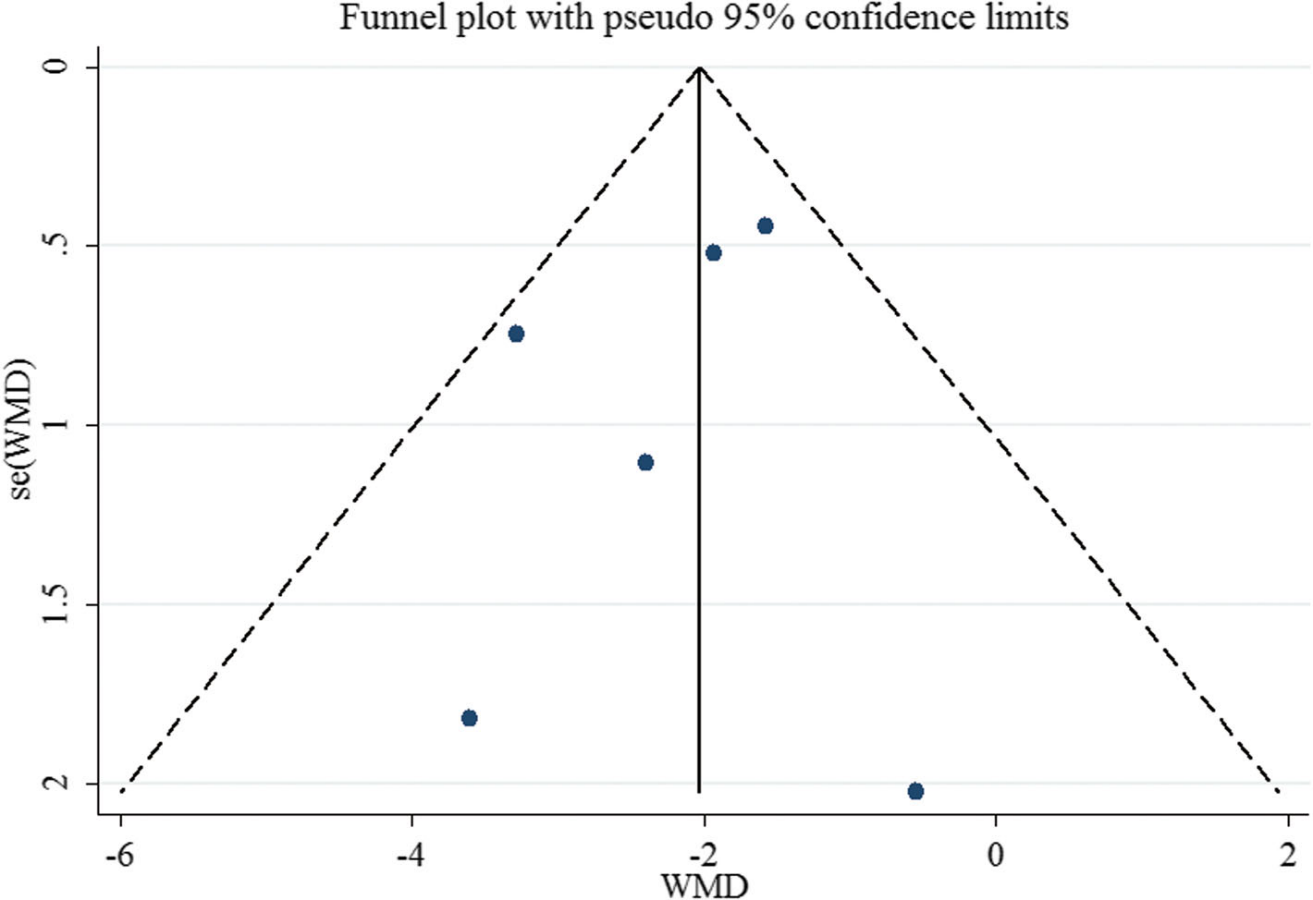
Funnel plot for publication bias analysis for vitamin D levels in CHB patients versus healthy controls

Funnel plot was relatively symmetrical, as displayed in Fig. 5. The result of Egger’s test also suggested no evidence of potential publication bias (*P* = 0.497).

#### Subgroup analysis

**Fig. 6:**
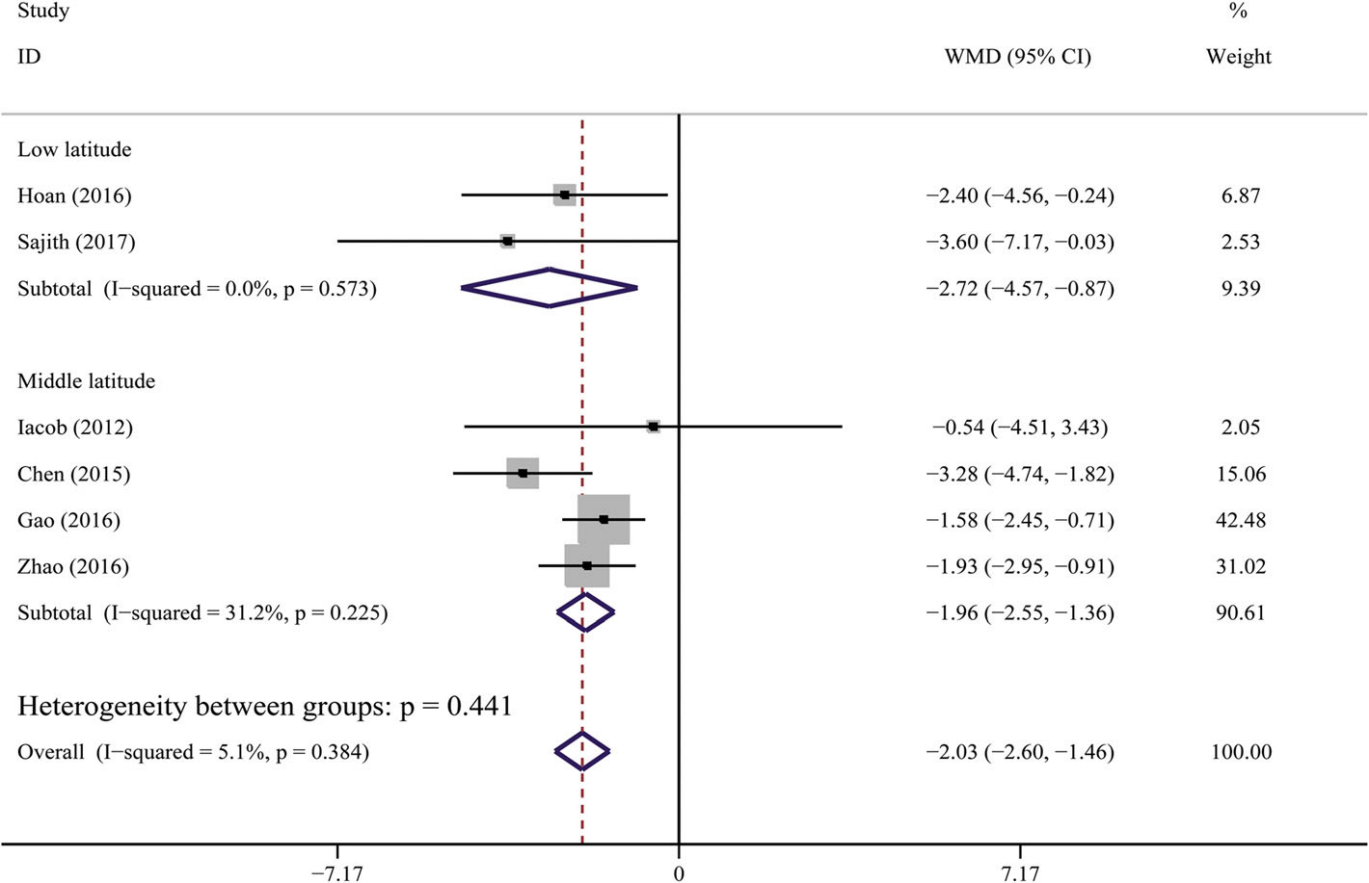
Subgroup analysis for comparisons of vitamin D levels in CHB patients versus healthy controls. Subtotals of low latitude and high latitude, and overall population

A gradient change in 25OHD level according to latitude was observed [10]. We therefore conducted subgroup analysis based on latitude of the geographical locations where the studies were performed. The vitamin D levels were also lower in CHB patients compared with healthy controls, irrespective of latitude (Fig. 6). This difference was more obvious in low latitude areas [pooled MD (95% CI) was − 2.72 ng/mL (− 4.57, − 0.87)].

#### Inverse correlation between vitamin D and HBV DNA levels

**Fig. 7:**
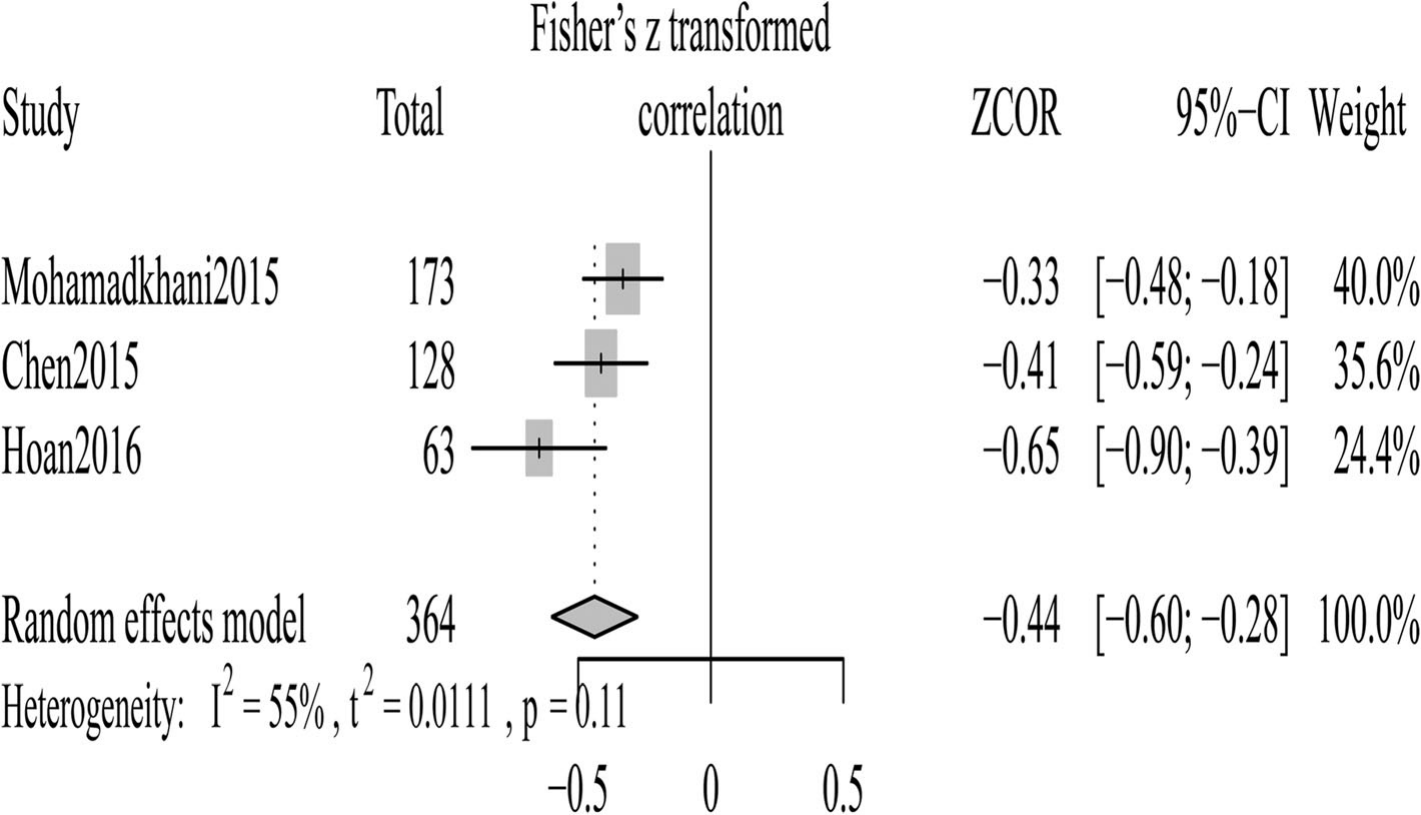
Forest plot of the association between vitamin D and HBV viral loads

We selected three studies [25–27] in which the relationship between vitamin D and HBV DNA levels was reported. When estimating a pooled correlation coefficient between serum 25OHD levels and HBV viral loads, a moderate heterogeneity between studies was observed, the random-effects model thereby was employed for the analysis. The result showed that the 25OHD levels was inversely correlated with HBV viral loads [summary Fisher’s Z value = − 0.44 (Cochran *Q* test *P* = 0.11, *I*^2^ = 55%) (Fig. 7); after turning to initial r, pooled r (95% CI) was − 0.41 (− 0.54, − 0.27)].

**Fig. 8:**
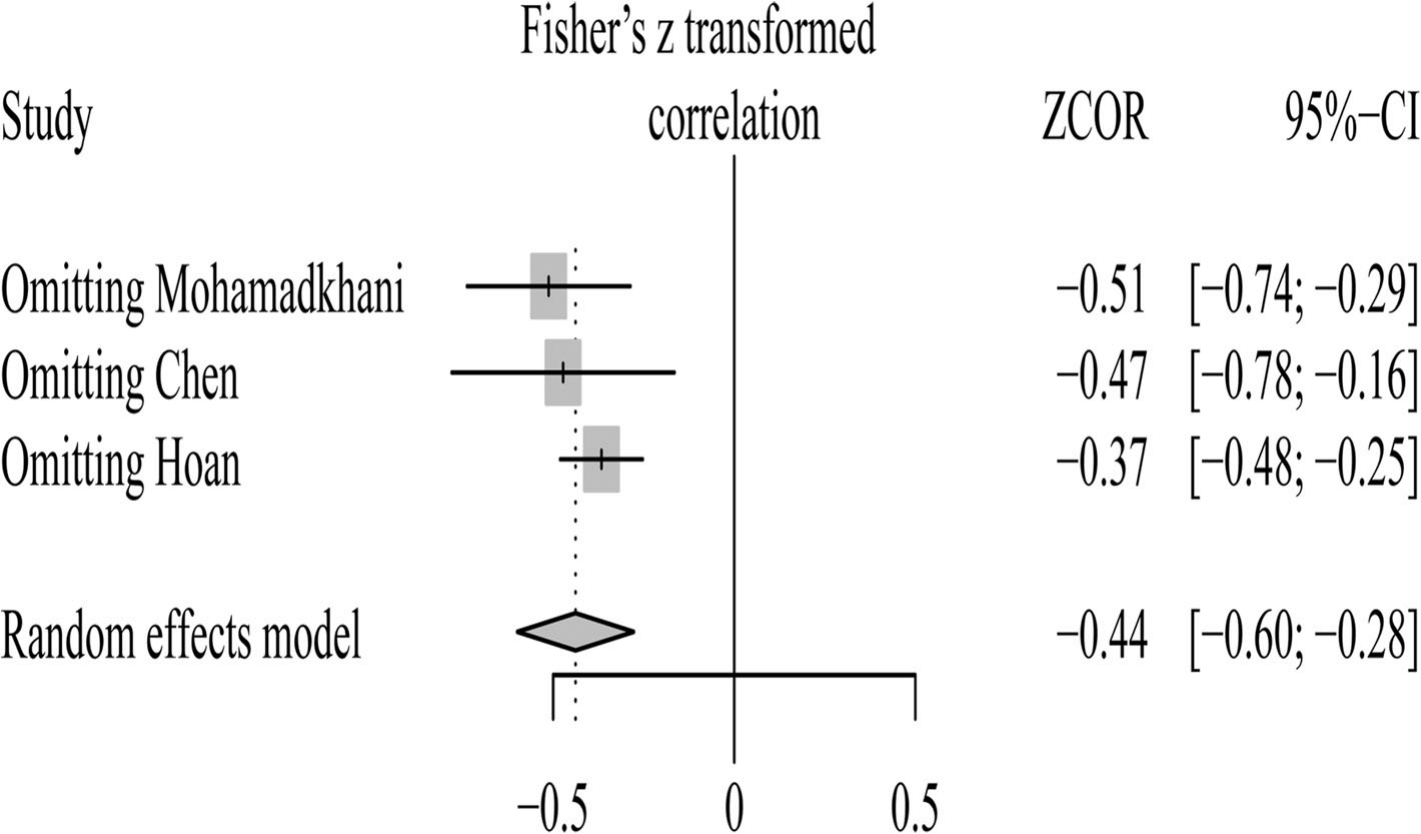
Sensitivity analysis for the association between vitamin D and HBV viral loads

Sensitivity analysis indicated that the study reported by Hoan and colleagues [27] was responsible for the heterogeneity. When this study was deleted, this heterogeneity reduced remarkably, with little influence on the analytical outcome [summary Fisher’s Z value = − 0.37 (Fig. 8); after turning to initial r, pooled r (95% CI) was − 0.35 (− 0.45, − 0.25)].

**Fig. 9:**
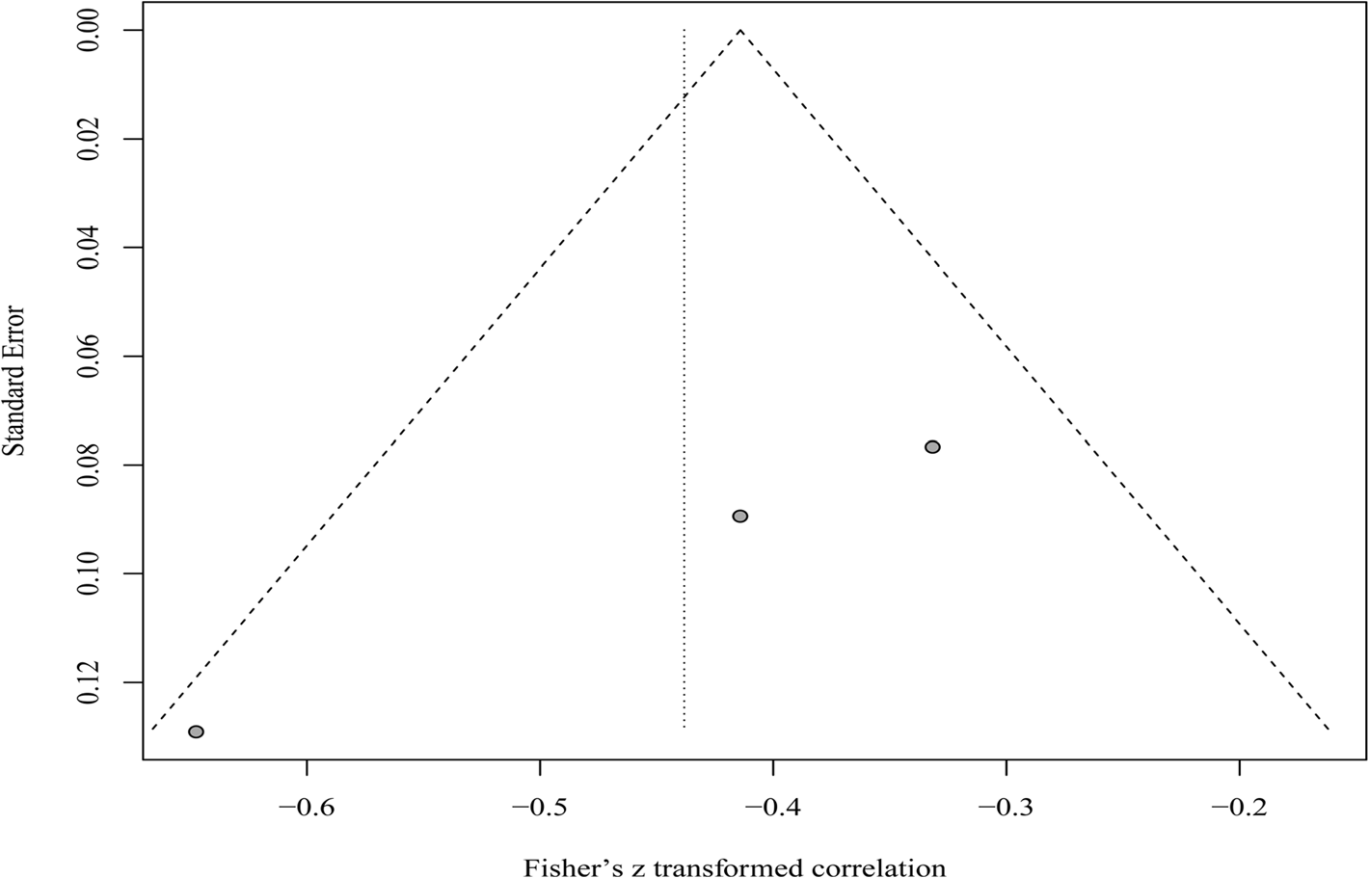
Funnel plot for publication bias analysis for the association between vitamin D and HBV viral loads

Funnel plot appeared asymmetric, indicating the possible publication bias (Fig. 9). However, due to the limited number of studies, Egger’s test cannot be conducted.

## Discussion

This meta-analysis evaluated the differences of vitamin D levels in CHB patients compared with healthy population and its correlation with HBV DNA levels. The key findings from our study are: (1) a significantly lower vitamin D levels in CHB patients with a 2.03 ng/mL decrease of vitamin D levels, compared with healthy controls, (2) an inverse correlation between serum 25OHD levels and HBV viral loads.

Several explanations are possible for the low vitamin D levels in CHB patients. Firstly, liver is a pivotal organ in the activation of vitamin D. The 25-hydroxylation of vitamin D takes place in the liver to produce 25OHD. This process is mediated by the 25-hydroxylases, including the microsomal CYP2R1 and the mitochondrial CYP27A1 enzymes [7, 8]. In addition, vitamin D-binding protein (DBP), the major carrier protein of 25OHD in the circulation, is exclusively synthesized by the liver [7]. Both of the enzymes and DBP are implicated with liver function. Thus the liver dysfunction of CHB patients could be a potential factor that contributed to the low vitamin D levels in these patients. Secondly, sunlight is an important determinant of vitamin D status via its initial generation in the skin [10]. Previous study [32] suggested that CHB patients had significantly lower physical activity levels than population norms, which may lead to a limited sunlight exposure and thereby a decreased vitamin D levels. Thirdly, a recent study [29] found that CYP24A1, an enzyme for degrading 25OHD and 1,25(OH)_2_D, was significantly upregulated in CHB patients when compared with healthy controls. Therefore, the imbalance of production and degradation of vitamin D could result in the vitamin D deficiency in CHB patients.

Subgroup analysis showed that the vitamin D levels were also lower in CHB patients compared with health controls, irrespective of latitude and this difference was more obvious in low latitude areas. In fact, several reviews found high prevalence of vitamin D deficiency worldwide, including countries of low latitude [33]. As for the Sajith’s study which was performed in low latitude country (India) [30], the decrease of vitamin D levels reached up to 3.60 ng/mL (most significant among included articles) in CHB patients compared with health controls (Fig. 6). Earlier studies showed that the vitamin D status is particularly poor in India, despite being a tropical country [34, 35]. And the possible reasons include inadequate direct sunlight exposure (due to the traditional, conservative pattern of clothing, etc.), and food habits, all of them potentially hamper the photosynthesis of vitamin D in the body [35–38].

It has been well established that host immune response significantly influence the pathogenesis of HBV infection [39, 40]. A weak immune response to HBV was found in chronic HBV infection [41]. Recent studies have shown that vitamin D has an important role in modulating both innate and adaptive immunity [7, 8, 27]. Various immune cells such as macrophages, B cells, T cells and antigen-presenting cells express VDR [7, 26]. And those CD8^+^ lymphocytes, the determining immune cells for HBV clearance, express the highest concentrations of VDR [42]. He et al [43] suggested that serum vitamin D levels have an obvious effect on the cellular immunity in CHB patients treated with interferon. The titer of HBV DNA decreased with the increase of serum vitamin D at baseline, and a higher level of serum 25OHD paralleled to a better virological response. Our meta-analysis demonstrates a negative relationship between vitamin D levels and HBV viral loads, though the number of relevant studies was only three. All these evidences indicate an important role of vitamin D in the immune response to HBV and even HBV replication itself. However, only three articles were enrolled and heterogeneity was found in our analysis. Therefore, the results should be interpreted with caution and more studies are needed to reach a robust conclusion in future. Also, it is still premature to recommend vitamin D supplementation for patients with CHB aiming to increase virological response.

In addition, vitamin D deficiency seems to be associated with disease progression in patients with chronic HBV infection. Wong et al [44] observed that serum 25OHD level was inversely correlated with Model for End-Stage Liver Disease score in CHB patients. And lower 25OHD level was an independent factor of long-term adverse outcomes. However, the exact cause of this relationship is far from clear. And meta-analysis in this regard is temporarily unavailable due to the limited information of relevant studies.

There were still several limitations in our meta-analysis. The major limitation is that the number of included articles was relatively small, which inevitably influence the stability of summarized results. Second, the CHB patients in our analysis was somehow underrepresented as one study only involved women, one only involved men and most included studies were from Asia. Third, the studies included in this work lacked information on either one or more characteristics (such as sampling season, gender, HBV genotype, HBeAg status), we could not perform stratified analysis by these items, the correspondingly influences on 25OHD level failed regretfully to be assessed and await more studies in future. Finally, the extraskeletal effects of vitamin D deficiency has been severely challenged, even raise the question about the real normal level of serum vitamin D in healthy population [45, 46]. Hence, it remains to be determined whether vitamin D has an impact on the HBV replication and the outcome of HBV infection.

## Conclusions

The current meta-analysis demonstrated a significant lower vitamin D levels in CHB patients compared with healthy people and an inverse correlation between serum vitamin D levels and HBV viral loads. Considering the limitations of the present study, further well-designed, comprehensive researches are needed to address the remaining issues.

## Additional files

Additional file 1: Data S1. PRISMA 2009 checklist. (DOC 74 kb)

Additional file 2: **Figure S1.** Conversion formulas. (TIF 34 kb)

## Data Availability

All data and materials were presented within the manuscript and additional supporting files.

## Abbreviations

1,25(OH)_2_D: 1,25-dihydroxyvitamin D
25OHD: 25-hydroxyvitamin D
CHB: Chronic hepatitis B
CHC: Chronic hepatitis C
CI: Confidence intervals
DBP: Vitamin D-binding protein
HBV: Hepatitis B virus
HCC: Hepatocellular carcinoma
MD: Mean difference
NAFLD: Non-alcoholic fatty liver disease
VDR: Vitamin D receptor
